# HMGA2 promotes nasopharyngeal carcinoma progression and is associated with tumor resistance and poor prognosis

**DOI:** 10.3389/fonc.2023.1271080

**Published:** 2024-01-18

**Authors:** Xinting Ouyang, Kangxin Li, Jiaqi Wang, Weijian Zhu, Qiang Yi, Jinghua Zhong

**Affiliations:** ^1^ Gannan Medical University, Ganzhou, Jiangxi, China; ^2^ Department of Oncology, First Affiliated Hospital of Gannan Medical University, Ganzhou, Jiangxi, China

**Keywords:** nasopharyngeal carcinoma, HMGA2, invasion metastasis, tumor resistance, prognosis

## Abstract

Nasopharyngeal carcinoma (NPC), as one of the most prevalent malignancies in the head and neck region, still lacks a complete understanding of its pathogenesis. Presently, radiotherapy, concurrent chemoradiotherapy, and targeted therapy stand as the primary modalities for treating NPC. With advancements in medicine, the cure rates for nasopharyngeal carcinoma have been steadily increasing. Nevertheless, recurrence and metastasis persist as the primary reasons for treatment failure. Consequently, a profound exploration of the molecular mechanisms underlying the occurrence and progression of nasopharyngeal carcinoma, along with the exploration of corresponding therapeutic approaches, becomes particularly imperative in the quest for comprehensive solutions to combat this disease. High mobility group AT-hook 2 (HMGA2) is a pivotal protein capable of altering chromatin structure, regulating gene expression, and influencing transcriptional activity. In the realm of cancer research, HMGA2 exhibits widespread dysregulation, playing a crucial role in nearly all malignant tumors. It is implicated in various tumorigenic processes, including cell cycle regulation, cell proliferation, epithelial-mesenchymal transition, angiogenesis, tumor invasion, metastasis, and drug resistance. Additionally, HMGA2 serves as a molecular marker and an independent prognostic factor in certain malignancies. Recent studies have increasingly unveiled the critical role of HMGA2 in nasopharyngeal carcinoma (NPC), particularly in promoting malignant progression, correlating with tumor resistance, and serving as an independent adverse prognostic factor. This review focuses on elucidating the oncogenic role of HMGA2 in NPC, suggesting its potential association with chemotherapy resistance in NPC, and proposing its candidacy as an independent factor in nasopharyngeal carcinoma prognosis assessment.

## 1 Introduction

Nasopharyngeal cancer is an epithelial malignant tumor that occurs in the mucosal layer of the nasopharynx (1). It has the highest incidence among head and neck tumors and is associated with factors such as Epstein-Barr virus infection, environmental factors, and genetics. Currently, the pathogenesis of this disease remains unclear. WHO classifies nasopharyngeal cancer into three subtypes: keratinized, non-keratinized and basal. Worldwide, the keratinizing subtype accounts for only about 20% of cases and is rare in endemic areas such as southern China; the non-keratinizing type accounts for the vast majority (>95%) of cases in endemic areas and is almost always associated with EBV infection, making it the most common histological subtype of nasopharyngeal cancer in endemic areas. According to the 2011 Global Cancer Statistics, the distribution of nasopharyngeal cancer has distinct geographic and ethnic differences (2), with East and Southeast Asia accounting for more than 70% of the global incidence of NPC, followed by South-Central Asia (6.3%), North Africa (2.6%) and South Africa (2.4%) (3). The disease has a predilection for several ethnic groups, including Cantonese living in southern China, Bidayou in Borneo, and Inuit living in the Arctic (4, 5). The clinical manifestations of nasopharyngeal cancer patients mainly include nasal congestion, nosebleeds, tinnitus, hearing loss, headache, and enlarged cervical lymph nodes. Radiotherapy, concurrent chemoradiotherapy, and targeted therapy are the main treatment modalities for nasopharyngeal cancer. Currently, the treatment strategy for nasopharyngeal cancer patients is mainly based on AJCC/IUCC staging. Intensity-modulated radiation therapy (IMRT) is the standard treatment for early-stage nasopharyngeal cancer (stage I- II), with a local regional control rate of over 90%. However, at the time of diagnosis, about 70% of patients have already reached the middle and late stages, leading to poor prognosis. Concurrent chemoradiotherapy with cisplatin can significantly improve the prognosis of late-stage (stage III-IVb) patients (6–8). To date, the 5-year overall survival (OS) rate of early-stage nasopharyngeal cancer patients is as high as 94%, while the 5-year OS rate (73.7%) of advanced-stage (III and IV) NPC patients is significantly reduced. Therefore, Early detection, early diagnosis, and early treatment can significantly improve the cure rate of nasopharyngeal cancer.

## 2 Structure and function of HMGA2

**Figure 1:**
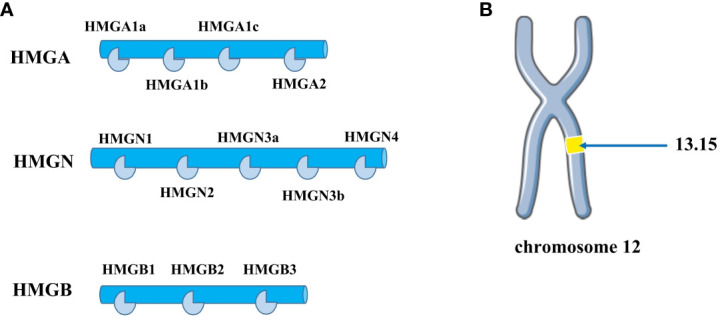
**(A)** The HMG protein superfamily consists of three subfamilies: HMGA, HMGB, and HMGN. The HMGA family includes four members: HMGA1a, HMGA1b, HMGA1c, and HMGA2. The HMGB family comprises three members: HMGB1, HMGB2, and HMGB3. The HMGN family contains several members, including HMGN1, HMGN2, HMGN3a, HMGN3b, and HMGN4. **(B)** HMGA2 is distributed on human chromosome 12q13-15.

High mobility group (HMG) proteins are non-histone chromatin proteins that are divided into three families based on their DNA-binding domains: HMGA, HMGB, and HMGN (9) (**Figure 1A**). Among them, HMGA proteins are the most studied and most relevant class. The HMGA family includes HMGA1a, HMGA1b, and HMGA2, and the HMGA1a and HMGA1b protein isoforms are selectively spliced from mRNA transcribed from the HMGA1 gene and are located on chromosome 6p21. HMGA2 is located on chromosome 12q13-15 (**Figure 1B**), up to 200 Kb, and contains 5 exons, each of which is encoded independently by the HMGA2 gene (10). In normal human tissues, HMGA2 encodes a 109-amino acid-containing intact protein product with a relative molecular weight of 12,000. HMGA2 itself is not transcriptionally active and regulates transcription mainly by altering chromatin structure (11), It binds to the AT-enriched region on the DNA of the regulated gene through the unique AT hook structure, causing the DNA to bend, stretch, and form a loop or untwin, thereby changing the chromatin structure and enhancing its transcriptional activity, also known as structural transcription factors (12). HMGA2 is also involved in the maintenance and functional regulation of DNA, including replication, recombination, transcription, and DNA repair (13).

HMGA2 is an important transcription factor that plays a key role in embryonic development. However, as embryonic development progresses, the expression of HMGA2 gradually decreases, and the expression of HMGA2 does not return to normal until embryo development is completed (14). The high expression of HMGA2 protein in embryos highlights its important role in development. HMGA2 levels are also significantly elevated during tumorigenesis, Fedele et al. (15) proposed that this is attributed to chromosomal rearrangements at chr12q13-15, disrupting the gene and consequently leading to aberrant protein expression. Furthermore, chromosomal breaks separate the open reading frame (ORF) of HMGA2 from its 3’ untranslated region (UTR), similarly resulting in the gene’s overexpression. More and more studies have shown that HMGA2 plays an important role in the development and progression of tumors by regulating tumor cell metastasis, epithelial-mesenchymal transition (EMT), and stemness of cancer stem cells (16–20). In past studies, abnormally high expression of HMGA2 has been observed in a variety of human cancers, such as esophageal squamous cell carcinoma (19),breast cancer (21), lung cancer (19), Thyroid cancer (22), melanoma (23), Colon cancer (24), Ovarian cancer (25, 26), bladder cancer (17) and other malignant tumors. Research indicates that HMGA2 accelerates cancer progression by activating multiple pathways. In this review, our emphasis is primarily on highlighting the oncogenic role of HMGA2 in nasopharyngeal carcinoma.

## 3 HMGA2 promotes the malignant progression of nasopharyngeal carcinoma

It has been found that HMGA2 is abnormally high in nasopharyngeal carcinoma. Liu et al. (27) downloaded data from the GEO database for analysis to study the expression of HMGA2 in nasopharyngeal carcinoma cell lines and tissues and found that HMGA2 was highly expressed in nasopharyngeal carcinoma cell lines and tissues. They further found that the expression level of HMGA2 in nasopharyngeal cancer tissues was significantly higher than that in normal nasopharyngeal tissues, and the expression of HMGA2 protein in nasopharyngeal cancer tissue samples was also significantly increased compared with nasopharyngeal tissue samples.

### 3.1 HMGA2 promotes the proliferation of nasopharyngeal carcinoma cells

HMGA2 promotes the proliferation of nasopharyngeal carcinoma cells by regulating the cell cycle. The cell cycle refers to the period from the beginning of cell division to the termination of DNA replication. In this process, cells reach a stable homeostasis through continuous growth, replication of genetic material, and cell division. A complete cell cycle process can be divided into 4 phases: G1 phase, S phase, G2 phase, and M (mitosis) phase, each of which is strictly regulated by related genes. Suski et al. (28) indicated that abnormal cell cycle regulation is common to all tumors, is present in almost all tumor types, and is the driving force of tumorigenesis. Therefore, anything that affects the cell cycle has the potential to lead to the development of tumors.

Studies have shown that HMGA2 can act as a regulator of cell proliferation, and its expression is increased in many types of human tumor tissues (29). Upregulation of HMGA2 expression can accelerate cell cycle progression and promote cell proliferation, while inhibition of HMGA2 expression can arrest cell cycle progression and lead to blocked cell proliferation. This may occur by direct binding of HMGA2 to cyclinA2’s cyclinA2-reactive elements, displacing the p120E4F-containing complex from cyclinA2, thereby inducing cyclinA2 expression and accelerating cell cycle progression (30, 31). In addition, Shaulian and Karin et al. (32) found that the transcription factor activating protein-1 (AP1) complex composed of Jun proteins (JUN, JUNB, and JUND), FOS proteins (FOS, FOSB, and FRA1), and FRA2 members is critical in the regulation of cell proliferation. Interestingly, Vallone et al. (33) found that in HMGA2-deficient cells, the expression of JUNB protein and FRA1 protein was completely inhibited, resulting in blocked cell proliferation. Conversely, when HMGA2 is abnormally highly expressed, these two proteins are correspondingly elevated, thereby promoting cell proliferation. Gao et al. (34) found that high expression of HMGA2 significantly accelerated the cell cycle process and promoted cell proliferation, while knockdown of HMGA2 expression caused the cell cycle to stagnate at a certain stage and inhibit cell proliferation. It can be seen that HMGA2 plays an important role in the regulation of the cell cycle, and excessive or low expression can disrupt the normal cell cycle, which in turn leads to the occurrence of malignant tumors. But it also brings us new thinking, and the development of new targeted drugs against HMGA2 expression is expected to be used to delay tumor progression, which provides clinicians with a new therapeutic direction. Although there are currently no targeted drugs for HMGA2, in previous clinical trials, we found that inhibiting the expression of HMGA2 with antisense oligonucleotide-modifying enzymes can effectively inhibit the proliferation and malignant transformation of tumor cells. Therefore, targeted regulation of HMGA2 expression may be a promising approach for future cancer treatments.

### 3.2 HMGA2-mediated angiogenesis promotes tumor metastasis

Tumor metastasis refers to tumor cells detaching from the primary site and migrating to a new site, invading the extracellular matrix, interacting with the extracellular matrix and surviving, then entering the blood vessels from the blood vessels, surviving in the blood, extravasating from the blood vessels, planting and growing in the target organ to form metastatic nodules (35). During metastasis, tumor cells need to cross the vascular barrier, which is a critical step in the metastasis process (36, 37). According to the literature, HMGA2 can promote angiogenesis in tumor tissues, which not only provides nutrients to tumor tissues but also facilitates the extensive metastasis of tumor cells. Sakata et al. (38) found *in vitro* that HMGA2 mainly affects the formation of tumor microenvironment by regulating angiogenesis-related genes, accelerating the angiogenesis process, and then affecting the vascular permeability of tumors, and the increased permeability of neovascularization can promote the invasion and metastasis of tumor cells.

In the study of nasopharyngeal carcinoma, Li et al. (39)found that the exosomal HMGA2 protein of EBV-positive nasopharyngeal carcinoma cells can induce the formation of a premetastatic microenvironment mediated by vascular leakage, thereby promoting the metastasis of nasopharyngeal carcinoma. At the same time, they also found that the exosomal HMGA2 protein of EBV-positive nasopharyngeal carcinoma cells can be delivered to endothelial cells, disrupting the integrity of endothelial junctions, thereby increasing vascular permeability, disrupting vascular barriers, and achieving distant metastasis of tumors. At the same time, through the analysis of a large number of clinical research data, the researchers found that serum exosome HMGA2 has unique advantages in predicting the metastatic potential of nasopharyngeal carcinoma, and it can be used as an effective non-invasive biomarker to detect the metastatic potential of nasopharyngeal carcinoma patients and provide a reference value for their prognosis evaluation. Therefore, serum exosome HMGA2 can be used as a promising prognostic biomarker and therapeutic target, thereby providing a better diagnosis and treatment strategy for patients with nasopharyngeal carcinoma. However, considering the issues of convenience and accuracy, more research is needed to find a standard and effective clinical method for detecting HMGA2 expression.

### 3.3 HMGA2-mediated EMT promotes the invasion and metastasis of nasopharyngeal carcinoma

Epithelial-mesenchymal transformation (EMT) refers to the biological process by which epithelial cells are programmed to transform into cells with a mesenchymal phenotype, which plays an important role in embryonic development, adult tissue regeneration, wound healing, and fibrosis (40, 41). The prominent changes characterized by EMT are the downregulation of epithelial markers such as E-cadherin and the upregulation of mesenchymal markers such as Vimentin (42), which are closely related to cancer cell invasion and metastasis. EMT is one of the important steps for cancer cells to gain invasion and metastasis, which can enhance the mobility, aggressiveness, self-renewal, and resistance of tumor cells, thereby promoting tumor cell invasion and metastasis (43, 44).

It has been reported that the EMT process is reflected in various types of cancer, including breast cancer (45), lung cancer (46), ovarian cancer (47), prostate cancer (48), and liver cancer (49). Numerous studies have demonstrated that HMGA2 expression is inextricably linked to the EMT process (50–53). Subsequently, it has been shown that HMGA2 regulates the expression of EMT transcription factors by binding to the abundant specific AT sequences in DNA and changing the conformation of chromatin (54), thereby enhancing tumor aggressiveness. In the study of Mansoori et al. (55), it was mentioned that high expression of HMGA2 can promote the expression of interstitial markers such as Vimentin and decrease the expression of epithelial markers such as E-cadherin, thereby increasing the chance of invasion and metastasis of malignant tumors. In the study of nasopharyngeal carcinoma, some researchers found that the expression of HMGA2 in nasopharyngeal carcinoma tissues was higher than that in normal tissues, and the differential expression of related proteins such as HMGA2 and EMT in nasopharyngeal carcinoma tissues and normal tissues suggested that HMGA2 and EMT and other related proteins played a potential role in the carcinogenesis of nasopharyngeal masses. Xia et al. (56) found that the expression levels of HMGA2 and EMT were associated with the progression and metastasis of nasopharyngeal carcinoma. At the same time, Wu et al. (57) experimentally found that the knockdown of HMGA2 inhibited the migration, invasion, and epithelial-mesenchymal transition of nasopharyngeal carcinoma cells. These results suggest that HMGA2 can mediate the EMT pathway to promote the invasion and metastasis of nasopharyngeal carcinoma.

### 3.4 HMGA2 is involved in several pathways of the EMT process

**Figure 2:**
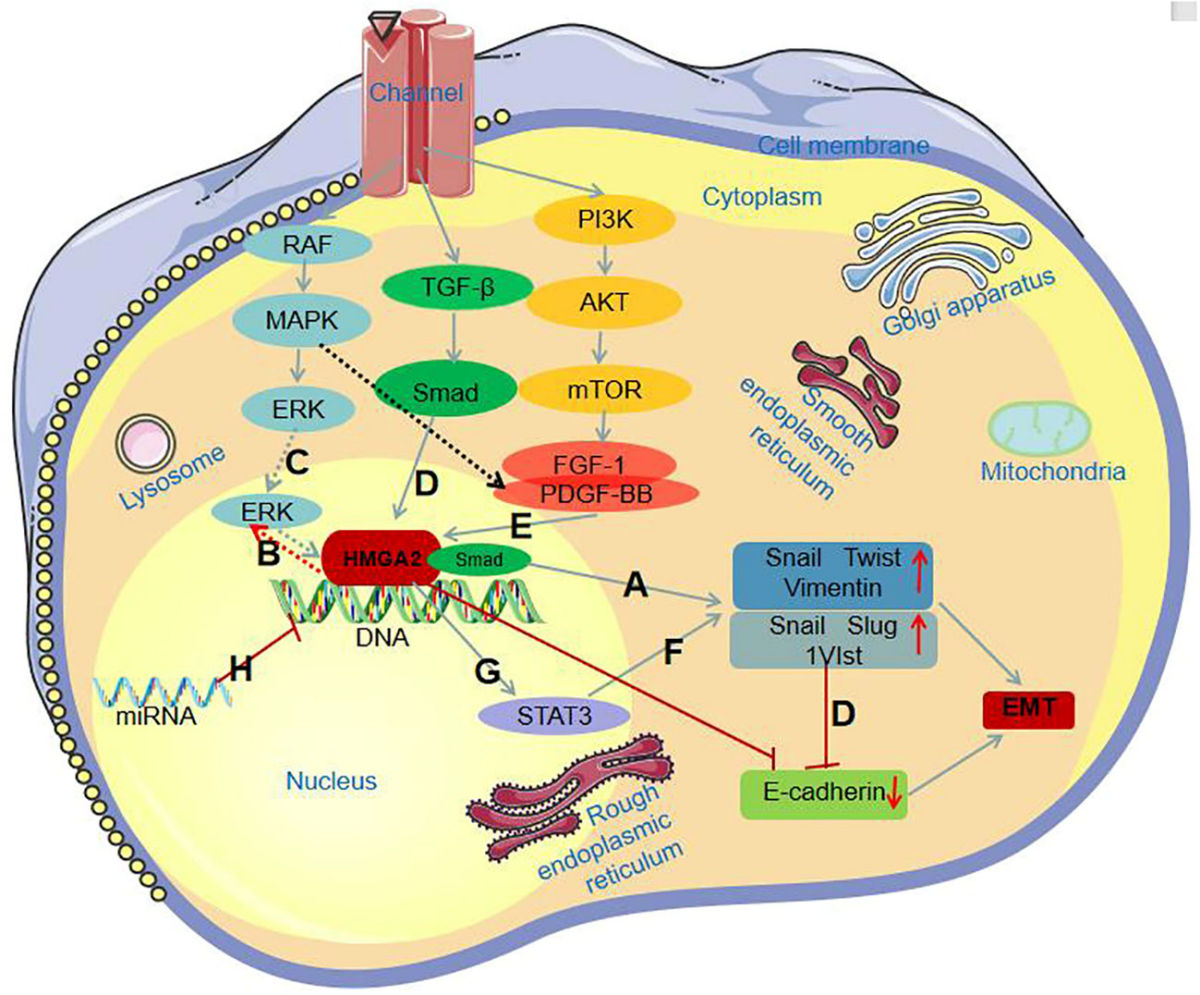
HMGA2 involvement in the EMT process through different signaling pathways. **(A)** HMGA2 induces the EMT process by increasing the expression of mesenchymal markers; **(B)** HMGA2 overexpression increases P-ERK levels; **(C)** HMGA2 induces the EMT process through the MAPK/ERK pathway; **(D)** Smad binds with HMGA2, collectively upregulating Snail, Slug, and 1V1st, leading to the downregulation of E-cadherin expression; **(E)** FGF-1 and PDGF-BB induce HMGA2 expression; **(F)** Activation of STAT3 expression increases the expression of mesenchymal markers; **(G)** HMGA2 induces the expression of STAT3; **(H)** HMGA2 is subject to reverse regulation by miRNAs.

They also found that HMGA2 plays an important role in the EMT process, and it can regulate intracellular signaling, thereby influencing the behavior and biological function of the cell. Mansoori et al. (55) studied the function of HMGA2 and found that it participates in the process of EMT through several signaling pathways, such as MAPK/ERK, TGF-β/Smad, PI3K/AKT/mTOR, NFkB, and STAT3, as well as miRNA expression regulation. In addition, it has been reported that HMGA2 can induce EMT in tumor cells by interfering with the cell cycle (58–60). HMGA2 primarily engages in the EMT process through the following pathways:

MAPK/ERK pathway:Hawsawi et al. (61), through immunofluorescence and protein blot analyses, discovered that HMGA2 induces the Epithelial-Mesenchymal Transition (EMT) process by upregulating the expression of mesenchymal markers such as Snail, Twist, and Vimentin (**Figure 2A**). Further investigations revealed that HMGA2 overexpression increases the levels of phosphorylated ERK (P-ERK) (**Figure 2B**). The use of the MAPK inhibitor U0126 to suppress the MAPK signaling pathway counteracts HMGA2-mediated EMT and cell migration processes. Hawsawi’s experimental results unequivocally demonstrate that HMGA2 induces EMT and cell migration through the MAPK/ERK pathway (**Figure 2C**). TGF-β/Smad pathway:In the TGF-β signaling pathway, Smad is capable of upregulating the inhibitory transcription factors Snail, Slug, and 1V1st through HMGA2, leading to the downregulation of the epithelial marker E-cadherin (**Figure 2D**). Consequently, this triggers epithelial-mesenchymal transition (EMT) (62). PI3K/AKT/mTOR pathway:As is well-known, Fibroblast Growth Factor-1 (FGF-1) and Platelet-Derived Growth Factor-BB (PDGF-BB) receptors are downstream signaling factors of the MAPK and PI3K pathways. Tan et al.’s study (63) indicates that FGF-1 and PDGF-BB can induce the expression of HMGA2 (**Figure 2E**). Conversely, inhibiting these receptors using corresponding inhibitors suppresses the expression of HMGA2. This suggests the crucial role of PI3K/AKT/mTOR signaling activation in the HMGA2-mediated Epithelial-Mesenchymal Transition (EMT) process.STAT3 pathway:According to available knowledge, STAT3 serves as a transcription factor for Twist and Snail, and the activation of STAT3 expression can increase the expression of mesenchymal markers (**Figure 2F**) (64). Additionally, HMGA2 induces the expression of STAT3 (**Figure 2G**). Aberrant expression of STAT3 increases Snail expression while reducing E-cadherin expression, thereby promoting the Epithelial-Mesenchymal Transition (EMT) process.miRNA Expression Regulation Pathway : Previous studies indicate that HMGA2 is subjected to reverse regulation by various microRNAs (miRNAs) (**Figure 2H**). The UTR structure within HMGA2 harbors numerous target sites, making it susceptible to targeted regulation by a variety of miRNAs. Mansoori et al. (50) discovered that stable induction of miR-330 expression leads to a reduction in HMGA2 expression, subsequently inhibiting Snail1 expression and increasing E-cadherin expression in cells, ultimately suppressing the Epithelial-Mesenchymal Transition (EMT) process.

## 4 HMGA2 promotes tumor drug resistance

Tumor drug resistance refers to the phenomenon that tumor cells can still survive and proliferate after the application of antitumor drugs. The presence of cancer stem cell populations in tumor tissue increases the probability of chemoresistance. There are two possible scenarios for tumor resistance, including intrinsic resistance and acquired resistance after chemotherapy (21). Secondary resistance of tumor cells to chemotherapy drugs is a major problem that needs to be solved urgently in clinical practice, which has attracted extensive attention from academia and society. More and more studies have shown that HMGA2 plays a very important role in the formation and development of drug resistance in tumor cells. It has been suggested that HMGA2 can promote drug resistance through intrinsic resistance and induce cancer stem cell populations. In the study of pancreatic cancer, Alfarouk et al. (65) found that HMGA2 is a downstream target gene of let-7a, which regulates the proliferation and metastasis of pancreatic cancer cells and increases chemosensitivity to gemcitabine. In addition, in *in vitro* and *in vivo* experiments on colorectal cancer, Li et al. (66) found that overexpression of HMGA2 enhanced chemoresistance to 5-fluorouracil (5-FU) by activating the Wnt pathway. Similarly, HMGA2 has also been shown to influence the process in resistance studies for nasopharyngeal carcinoma. Studies have found (67) that HMGA2 is involved in the regulation of cisplatin resistance, and the expression level of HMGA2 in nasopharyngeal carcinoma cell lines is positively correlated with the degree of cisplatin resistance, and inhibiting the expression or function of HMGA2 can increase the sensitivity of cisplatin to nasopharyngeal carcinoma cells. In addition, some studies have reported the possibility that HMGA2 is involved in modulating the resistance mechanism of other anti-nasopharyngeal cancer drugs, such as docetaxel, and resistance to targeted therapy drugs. However, it should be noted that the above studies on nasopharyngeal carcinoma are *in vitro* or mouse model experiments and their role in clinical treatment needs to be further verified. Therefore, HMGA2 has been shown to increase drug resistance in a variety of malignant tumors, including nasopharyngeal carcinoma, thereby affecting the therapeutic effect of tumors, and these research results provide new ideas and targets for anti-drug resistance to malignant tumors.

It is generally accepted that autophagy is essential in the process of drug resistance in a variety of cells, such as glioma in solid tumors, osteosarcoma, and acute myeloid leukemia in non-solid tumors (68–72) Autophagy is an essential ability of cells to restore their energy balance during different nutrient supply periods (73), and it is an important part of a variety of cell biological functions, and its dysregulation is closely related to tumorigenesis, tumor-stromal interactions, and chemoresistance. In contrast to previous studies that HMGA2 can promote tumor resistance, Xu et al. (51)reported a paradoxical role in a study in which they showed that HMGA2 overexpression inhibited gefitinib resistance in non-small cell lung cancer by inhibiting autophagy. They found that HMGA2 was able to downregulate the expression of LC3B-II, a key marker of autophagy, and subsequently reduced gefitinib resistance by inhibiting autophagy. Therefore, the claim that HMGA2 promotes tumor drug resistance is not absolute, and more clinical trials are needed to confirm it. However, several studies have demonstrated a strong relationship between HMGA2 and cancer chemotherapy resistance, and we cannot completely dismiss this conclusion based on a single study.

## 5 HMGA2 is associated with poor prognosis of nasopharyngeal carcinoma

HMGA2 is significantly associated with poor prognosis in a variety of malignancies. Through the comparative analysis of tumor tissue and normal tissue, it was found that there was a significant correlation between the expression level of HMGA2 and different tumor types. By measuring the expression level of HMGA2, the prognosis of patients with different tumor types can be judged more accurately. Some studies have found a strong association between HMGA2 expression levels and patient survival time. This suggests that HMGA2 may be an important prognostic marker. In the melanoma study, Raskin et al. (23) found that HMGA2 overexpression was significantly associated with a decrease in overall survival (OS) in melanoma patients, suggesting that high expression of HMGA2 was associated with poor prognosis in patients with malignant melanoma. Li et al. (74) found that high expression of HMGA2 is strongly associated with tumor metastasis and prognosis, and they found that HMGA2 is an independent prognostic factor in lung cancer through Cox multivariate analysis, which was also confirmed by Gao et al. (75).

Similarly, studies have shown that high expression of HMGA2 is closely related to poor prognosis in patients with nasopharyngeal carcinoma. Liu et al. (27) collected the clinical data of 116 patients with nasopharyngeal carcinoma with prognostic information and analyzed these data, and found that HMGA2 expression was significantly correlated with the overall survival time of patients with nasopharyngeal carcinoma, and the overall survival time of patients with high HMGA2 expression was significantly shorter than that of patients with low HMGA2 expression level, suggesting that high HMGA2 expression was associated with poor prognosis of patients with nasopharyngeal carcinoma. Xia et al. (56) found that high expression of HMGA2 and N stage were independent prognostic factors for nasopharyngeal carcinoma, indicating that high expression of HMGA2 and N stage were associated with poor prognosis in patients with nasopharyngeal carcinoma, suggesting that HMGA2 can be used as an effective prognostic biomarker for nasopharyngeal carcinoma. At the same time, the discovery of this prognostic biomarker may have an effect on molecularly targeted therapy for nasopharyngeal carcinoma. However, more prospective studies are needed to validate this.

## 6 Summary and prospects

**Figure 3:**
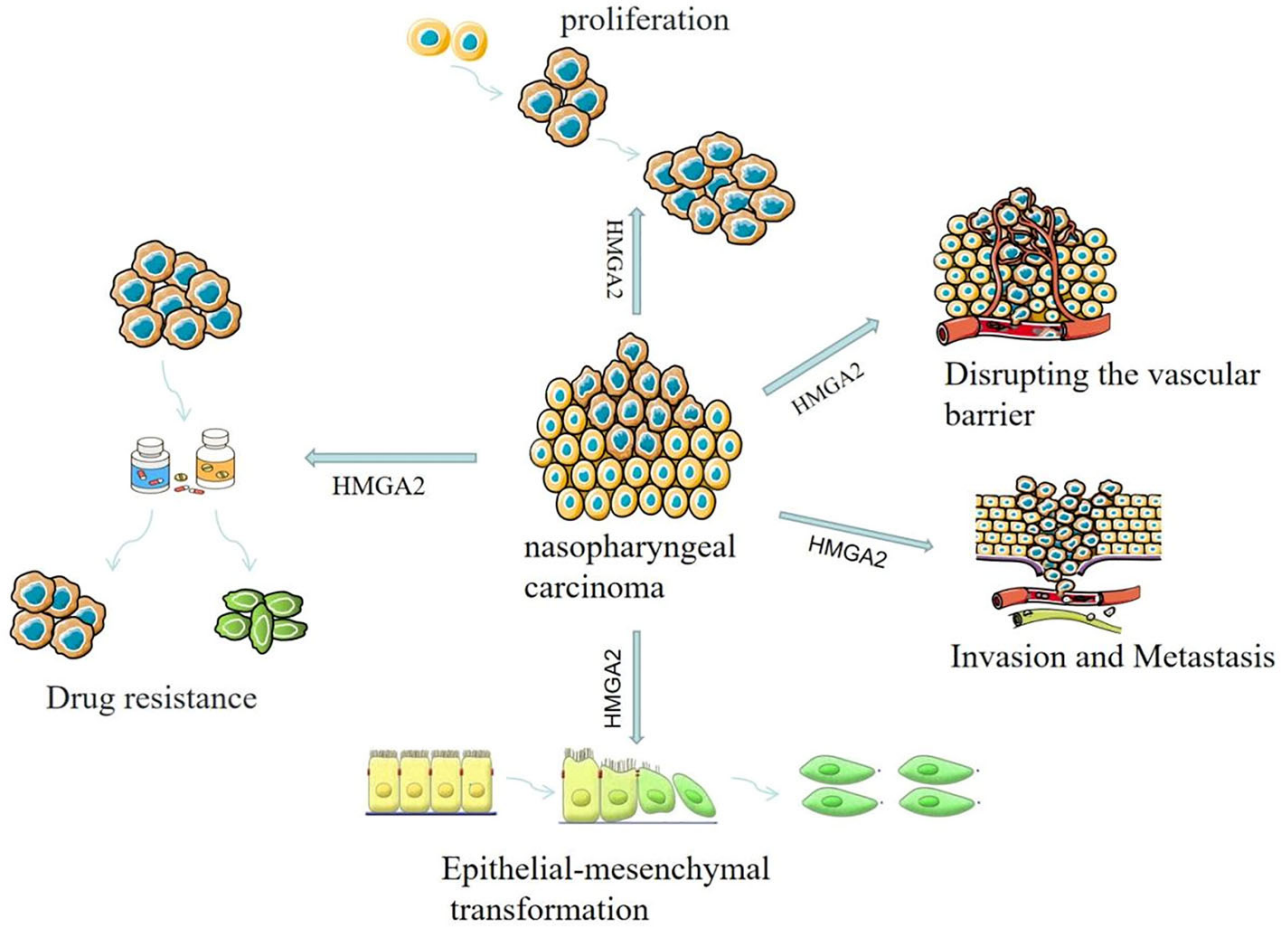
Role of HMGA2 in the occurrence and development of nasopharyngeal carcinoma.

In summary, HMGA2 is a key regulator of the malignant progression of nasopharyngeal carcinoma (**Figure 3**). HMGA2 exhibits abnormally high expression in nasopharyngeal carcinoma cell lines and tissues. It can regulate the cell cycle, promote cell proliferation, induce epithelial-mesenchymal transition, promote angiogenesis, and increase vascular permeability. Consequently, HMGA2 contributes to the malignant progression of nasopharyngeal carcinoma (**Table 1**). At the same time, HMGA2 can promote drug resistance in tumor cells, which will provide a new theoretical basis and strategy for clinical anti-tumor drug therapy, but more experiments are still needed to verify this. In addition, HMGA2 is closely related to the malignancy of nasopharyngeal carcinoma and is an independent prognostic factor for nasopharyngeal carcinoma, which can be used as an important prognostic molecular marker for nasopharyngeal carcinoma, which will provide new ideas for the early diagnosis and effective treatment of nasopharyngeal carcinoma. Due to its unique biological properties, HMGA2 plays an important role in tumorigenesis, development, invasion, and metastasis. Thus, HMGA2 holds promise as a significant biomarker for the early diagnosis and prognostic assessment of cancer. However, current research still faces several limitations. For instance, the practical application value of HMGA2 in early cancer diagnosis and prognostic assessment requires further validation. The lack of effective clinical methods to detect HMGA2 expression and the absence of uniform standards to gauge its prognostic value. Additionally, the detailed mechanisms of HMGA2 in conferring resistance in nasopharyngeal carcinoma and its specific regulatory role in tumor stemness pathways remain insufficiently explored, necessitating further in-depth investigation. Addressing these limitations will be crucial for the future direction of research in this field.

**Table 1:** The Function and significance of HMGA2 in nasopharyngeal carcinoma and related mechanisms.

Gene	Cancer	Function and significance	Related mechanisms
HMGA2	Nasopharyngeal carcinoma	Regulating the cell cycle to promote the proliferation of nasopharyngeal carcinoma cells.	This may occur by direct binding of HMGA2 to cyclinA2’s cyclinA2-reactive elements, displacing the p120E4F-containing complex from cyclinA2, thereby inducing cyclinA2 expression and accelerating cell cycle progression (30, 31).
		Regulating genes related to vascular generation, increasing vascular permeability, and accelerating distant metastasis of tumors. Participating in the Epithelial-Mesenchymal Transition process, promoting tumor invasion and metastasis.	HMGA2 protein can be delivered to endothelial cells, disrupting the integrity of endothelial junctions, thereby increasing vascular permeability, disrupting vascular barriers, and achieving distant metastasis of tumors (39) high expression of HMGA2 can promote the expression of interstitial markers such as Vimentin and decrease the expression of epithelial markers such as E-cadherin, thereby increasing the chance of invasion and metastasis of malignant tumors (55).
		Promoting tumor drug resistance. Associated with the prognosis of malignant tumors, it may be an independent factor indicating poor prognosis in nasopharyngeal carcinoma.	HMGA2 is involved in the regulation of cisplatin resistance, and the expression level of HMGA2 in nasopharyngeal carcinoma cell lines is positively correlated with the degree of cisplatin resistance, and inhibiting the expression or function of HMGA2 can increase the sensitivity of cisplatin to nasopharyngeal carcinoma cells (67). the overall survival time of patients with high HMGA2 expression was significantly shorter than that of patients with low HMGA2 expression level, suggesting that high HMGA2 expression was associated with poor prognosis of patients with nasopharyngeal carcinoma (27). high expression of HMGA2 and N stage were independent prognostic factors for nasopharyngeal carcinoma (56)

In conclusion, the close association of HMGA2 with the malignant progression, drug resistance, and adverse prognosis of nasopharyngeal carcinoma underscores its potential as a significant malignancy biomarker. This suggests that HMGA2 has the potential to become a crucial target for diagnosis, treatment, and prognostic assessment in nasopharyngeal carcinoma.

## Author contributions

XO: Formal analysis, Conceptualization, Investigation, Methodology, Software, Writing – original draft. KL: Formal analysis, Supervision, Investigation, Methodology, Writing – original draft. JW: Formal analysis, Investigation, Supervision, Software, Validation, Writing – review & editing. WZ: Formal analysis, Conceptualization, Methodology, Writing – original draft. QY: Conceptualization, Writing – original draft, Investigation, Software. JZ: Formal Analysis, Funding acquisition, Project administration, Resources, Supervision, Writing – review & editing.
